# Urinary Bladder Nephrogenic Adenoma: A Histopathological Case Report

**DOI:** 10.7759/cureus.62281

**Published:** 2024-06-12

**Authors:** Hristo Popov, Andreya Kirilova, Kristina Naydenova, Ekaterina Softova, George S Stoyanov

**Affiliations:** 1 General and Clinical Pathology, Forensic Medicine and Deontology, Medical University of Varna, Varna, BGR; 2 Pathology, Individual Medical Diagnostic Laboratory City Lab, Varna, BGR; 3 Pathology, Multiprofile Hospital for Active Treatment, Shumen, BGR

**Keywords:** urinary bladder, benign lesions, urology, pathology, nephrogenic adenoma

## Abstract

Nephrogenic adenomas are benign lesions that develop within the urinary tract. Most often developing within the urinary bladder, these lesions have a debatable etiopathogenesis, with hamartoma, rest hyperplasia, and transplantation of renal tubular cells being the most widely accepted ones. Nephrogenic adenomas develop more often in adult males, and predisposing factors for their development are prior urinary system injury, infection, or malignancy, with a subset of cases developing in renal transplant patients. Herein, we present a case of a male patient in his seventies who initially presented to our institution with urinary disturbances and was subsequently diagnosed with low-grade, non-invasive urothelial carcinoma. After treatment, the patient remained disease-free for a period of seven calendar years. The current presentation was due to dysuria, and bladder endoscopy revealed a ureteral stricture and two small exophytic lesions neighboring the location of the previously treated urothelial carcinoma. Histology revealed complex papillary architecture and cystic spaces lined by a monolayer of monomorphous epithelial cells with foci of hobnail appearance. The papillary stroma consisted of edematous fibrous tissue with hyperemic blood vessels and focal infiltration by inflammatory cells. Based on the histological findings, the diagnosis of nephrogenic adenoma was established.

## Introduction

First described in 1949 by Davies and later defined more accurately and coined as a term in 1950 by Friedman and Kuhlenbeck, nephrogenic adenoma is a rare and dubious medical condition [1-3]. While initially described as a hamartoma by Davies and later thought to be a benign tumor by Friedman and Kuhlenbeck, the biology of nephrogenic adenoma remains debatable. While some authors believe it to originate from embryonal and fetal remnants of renal tubular tissue residing within the urinary bladder wall and slowly undergoing hyperplasia, some depict a metaplastic mechanism for the transformation of native urinary bladder structures, with the most widely adopted theory for its development being the implantation one [4-6]. The implantation theory depicts the origin of nephrogenic adenoma from detacher renal tubular cells and whole structures that successfully implant themselves throughout the urinary system but predominantly within the bladder [4,6]. In support of this theory of origin is the fact that the majority of depicted cases are in patients with previous kidney injuries, such as infections, recurrent catheterization, and local bladder therapy after malignancies, and renal transplant patients [3-5,7]. Furthermore, most of the depicted cases are in adult individuals, with a significant male predominance [3,5,6]. Still, a subset of cases are depicted within the pediatric population [3,6].

Herein, we report a histopathological case of nephrogenic adenoma in a male patient with a previous medical history of bladder malignancy.

## Case presentation

A 77-year-old male presented to our institution seven years prior with complaints of painless gross hematuria. An endoscopy of the urinary bladder showed an exophytic, finely branched lesion measuring 1.5 cm on the lower aspect of the posterior bladder wall, which was resected transurethrally. Histology of the resected lesion showed low-grade, non-invasive (pTa) urothelial carcinoma for which intravesical therapy was initiated. The patient remained stable and without new complaints until the current presentation. Previous medical history was also significant for hypertension for the previous 20 years, under adequate medication control.

In the current presentation, the patient complained of frequent urination, with difficulties in fully emptying the bladder, sometimes accompanied by pain and a general decrease in urine stream. The ultrasound did not reveal hyperplasia of the prostate, and under suspicion for ureteral stricture, due to the previous medical history, a new bladder endoscopy was scheduled.

Bladder endoscopy under general anesthesia revealed a slight stricture in the upper third of the urethra, which was successfully dilated with a balloon. On the left lower posterior aspect of the bladder wall, neighboring the area of the previous resection, two exophytic lesions were noted with a diameter of 3 and 4 millimeters, respectively, which were successfully resected.

**Figure 1:**
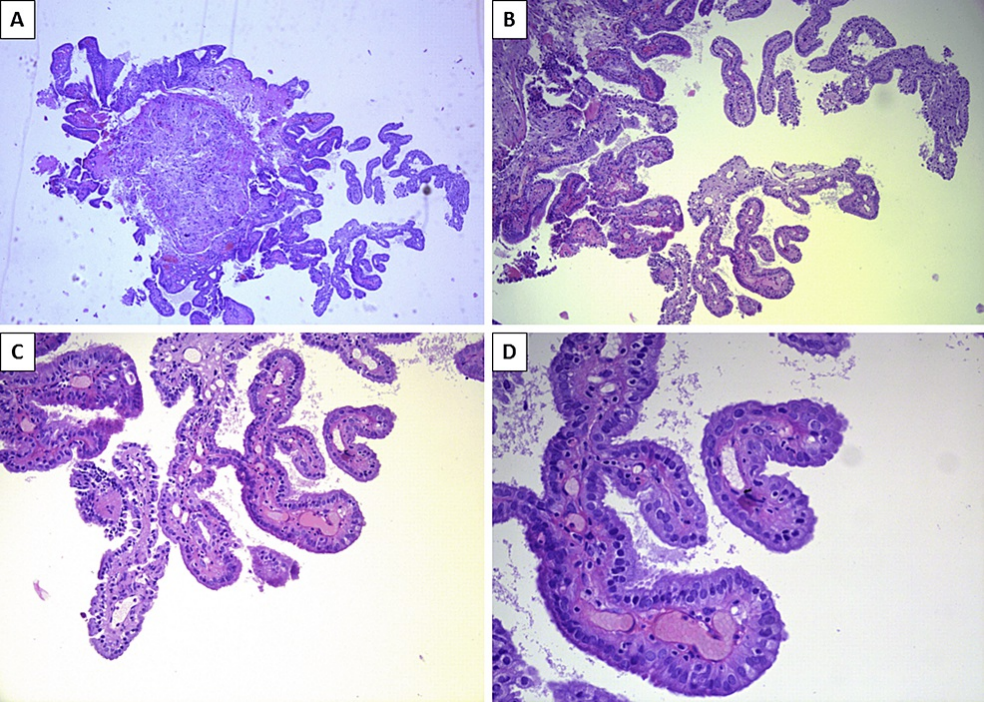
Histopathological appearance of nephrogenic adenoma A: complex papillary architecture, H&E stain, original magnification x40; B: branching and fusing papillae, H&E stain, original magnification x100; C: papillae with edematous stroma and hyperemic blood vessels, lined by a single layer of cylindrical and cubical cells, H&E stain, original magnification x200; D: papillae with edematous stroma, hyperemic blood vessels, scant lymphocytic inflammatory infiltration, covered by a single layer of monomorphous cylindrical cells, with nuclear hobnail appearance, H&E stain, original magnification x400 H&E: hematoxylin and eosin

Histopathology of the rested lesion revealed that the lesions had a complex papillary architecture, with branching and fusing papillae and areas of cystic spaces, both lined by a single layer of cylindrical, cubical and flat, monomorphic epithelial cells, albeit with foci of hobnail appearance (Figure 1). The stroma of the papillae consisted of fine and edematous fibrous tissue with hyperemic blood vessels and minimal focal infiltration by inflammatory cells - lymphocytes, plasma cells, and a few granulocytes, including eosinophils. One of the specimens had a section of preserved bladder mucosa that transitioned with the described complex papillary structures. As per the histological appearance of both lesions, the diagnosis of nephrogenic adenoma was established.

Following the endoscopic intervention, the patient's complaints were resolved. He has remained stable, without new or recurrent complaints, for one calendar year.

## Discussion

As already stated, nephrogenic adenomas are benign lesions with unclear etiopathogenesis - hamartoma, metaplasia, or implantation of normal tissue [3]. Whilst being benign, the diagnostic process in these cases is challenging due to most patients having, in one form or another, a previous or persistent urological condition [5]. Hence, the clinical diagnosis in these cases is most often that of malignant neoplasia development or recurrence.

While in our case, the endoscopic suspicion was that of urothelial carcinoma recurrence due to the patient's medical history, the size of the synchronous lesions was relatively small. Published cases depict sizes varying from several millimeters to a centimeter, as in our case, to upwards of five centimeters [8,9].

Furthermore, the papillary structure of the lesions may often sway the pathologist toward a malignant tumor, especially concerning the papillary growth pattern of the bladder native urothelial carcinoma. Furthermore, there are several variants and types of nephrogenic adenoma, with the one depicted in the present case - papillary being the most commonly depicted one. The second most commonly depicted one is tubular, where instead of papillae forming, the adenoma presents with tubular structures lined by the same epithelium; the tubules have a thickened, hyalinized basement membrane and are scattered within a blood vessel and inflammatory infiltrate-rich, fibrotic stroma; in cases when the tubules are cystically dilated, the type is referred to as tubulocystic [9,10]. Tubular and papillary patterns commonly coexist in the same case, and this pattern is depicted as mixed. In contrast, in our case, there were tubular structures consistent with the above-given description; however, they were too scarce to define the case as a mixed tubulopapillary nephrogenic adenoma. More exotic variants are flat nephrogenic adenomas wherein the adenoma epithelial cells line the bladder wall in a replacement pattern to the neighboring urothelium; this pattern often raises suspicion for urothelial atypia and is reported to be a common component of most types of nephrogenic adenoma [11]. Fibromyxoid nephrogenic adenomas present morphologically with scatter renal tubular cells, often flattened and spindle-shaped in background fibromyxoid stroma. This variant is extremely challenging when not mixed with more classical morphological types and requires the use of immunohistochemical markers to prove the renal tubular origin and exclude mesenchymal tumors or carcinosarcoma [8]. Probably the most challenging and concerning for malignancy type is the signet ring-like variant of nephrogenic adenoma, in which the growth pattern is predominantly that of tubular or tubulocystic with clear cell transformation of the epithelium and degenerative nuclear atypia resembling adenocarcinoma-like signet ring cells [6].

## Conclusions

Nephrogenic adenomas are benign lesions with unclear etiopathogenesis developing within the urinary system. They develop predominantly in adults with preexisting urological disease. They are a histopathological challenge due to the clinical suspicion of malignancy development or recurrence and their multiple histological variants, some of which are challenging to differentiate from urinary system-native and metastatic diseases and require a broad differential diagnosis. As seen in our case, wherein the nephrogenic adenoma developed in an elderly male with a history of urothelial carcinoma, the lesion may be incidental findings with a small size and indolent clinical symptoms, but in rare cases, both lesion size and symptoms may be striking.
